# Prevalence of Human Papillomavirus Infection by Number of Vaccine Doses Among US Women

**DOI:** 10.1001/jamanetworkopen.2019.18571

**Published:** 2019-12-27

**Authors:** Kalyani Sonawane, Alan G. Nyitray, Gizem S. Nemutlu, Michael D. Swartz, Jagpreet Chhatwal, Ashish A. Deshmukh

**Affiliations:** 1 Center for Healthcare Data, Department of Management, Policy, and Community Health, UTHealth School of Public Health, Houston, Texas; 2 Center for Health Services Research, Department of Management, Policy, and Community Health, UTHealth School of Public Health, Houston, Texas; 3 Center for AIDS Intervention Research, Clinical Cancer Center, Medical College of Wisconsin, Milwaukee; 4 Massachusetts General Hospital Institute for Technology Assessment, Harvard Medical School, Boston; 5 Department and Biostatistics and Data Science, UTHealth School of Public Health, Houston, Texas

## Abstract

This cross-sectional study investigates the prevalence of human papillomavirus (HPV) infection among US women who received between 0 and 3 doses of HPV vaccine.

## Introduction

More than a decade after the introduction of the human papillomavirus (HPV) vaccine in the United States, only 51.1% of adolescents have completed the vaccine series, while a greater number (68.1%) received at least 1 dose.^1^ The suboptimal series completion rate in the United States is partly attributable to the barriers, including unawareness of or forgetting the need for additional doses, lack of insurance coverage or health care professional recommendations, and less frequent contact with the medical system.^2,3^ To simplify the recommendations, trials are evaluating the efficacy of a single-dose regimen.^4^ In this study, we investigated HPV infection prevalence among women by number of vaccine doses received.

## Methods

This cross-sectional study analyzed National Health and Nutritional Examination Survey (NHANES) 2009 to 2016 data, which are a stratified multistage probability sample of the US population. Demographic characteristics and immunization history were self-reported and collected by trained interviewers during a home interview. Sexual behavior data were self-reported by participants in the Mobile Examination Center. Participants provided self-collected cervicovaginal swab specimens. The specimens were evaluated by polymerase chain reaction followed by type-specific hybridization. Details of the survey questionnaire, sample collection, and laboratory methods are available elsewhere.^5^

We identified women aged 18 to 26 years at the time of survey participation with nonmissing HPV vaccination and HPV test data. Nationally representative estimates for prevalence and the representative population counts were computed using NHANES sampling weights. The survey weight–adjusted Wald *F* test was used to examine the difference in the prevalence of HPV infection (4-valent vaccine types [HPV types 6, 11, 16, and 18]; cross-protection types [HPV types 31, 33, and 45]; and other high-risk types [HPV types 35, 39, 51, 52, 56, 58, 59, and 68]) by the number of doses received. The differences in predicted probability for HPV types 6, 11, 16, and 18 by number of vaccine doses and by the levels of risk factors were estimated using a multivariable logistic regression model. The model was adjusted simultaneously for age as a linear term, race/ethnicity, age at sexual debut, and lifetime number of male sexual partners. Statistical significance was tested at 2-sided *P* < .05. All analyses were performed with SAS software version 9.4 (SAS Institute) using SAS PROC SURVEY procedures, which included weight, cluster, and strata statements, to incorporate sampling weights and to adjust for the complex survey design.

This study was deemed exempt from review and requirements for patient informed consent by the institutional review board of the University of Texas Health Science Center owing to the use of publicly available and anonymized data. This study followed the Strengthening the Reporting of Observational Studies in Epidemiology (STROBE) reporting guideline.

## Results

The study sample included a total of 1620 women (mean [SE] age, 22.2 [0.1] years; 56.5% white), of whom 1004 were unvaccinated and 616 received at least 1 dose of HPV vaccine: 106 received 1 dose, 126 received 2 doses, and 384 received 3 doses.

Compared with unvaccinated women (prevalence of 12.5% [95% CI, 9.7%-15.3%]), infection with HPV type 6, 11, 16, or 18 was significantly less prevalent among women who received 1 dose (2.4% [95% CI, 0%-4.9%]), 2 doses (5.1% [95% CI, 0.8%-9.5%]), or 3 doses (3.1% [95% CI, 0.9%-5.3%]) of HPV vaccine (Table 1). There was no significant difference in prevalence for 1 dose vs 2 doses or 1 dose vs 3 doses. Differences were not statistically significant for cross protection (except for 2 doses vs unvaccinated and 1 dose vs 2 doses) and other high-risk HPV types.

**Table 1.** Prevalence of Genital HPV Infection Among HPV-Vaccinated and Unvaccinated Women Aged 18 to 26 Years, National Health and Nutrition Examination Survey, 2009-2016

Value	Vaccinated			Unvaccinated (n = 1004)
	1 Dose (n = 106)	2 Doses (n = 126)	3 Doses (n = 384)	
**HPV Types 6, 11, 16, 18 (4-Valent Vaccine–Type Infection)**				
No. with infection	4	7	14	111
Weighted				
Prevalence (95% CI), %	2.4 (0.0-4.9)	5.1 (0.8-9.5)	3.1 (0.9-5.3)	12.5 (9.7-15.3)
No. with infection/total No.	22 459/924 276	61 684/1 200 402	121 940/3 919 600	1 179 961/9 440 387
*P *value vs 2 doses^a^	.12			
*P* value vs 3 doses^a^	.70	.40		
*P* value vs unvaccinated^a^	<.001	.003	<.001	
**HPV Types 31, 33, and 45 (Cross-Protection Types)**				
No. with infection	11	3	22	57
Weighted				
Prevalence (95% CI), %	10.7 (3.5-18.0)	2.8 (0.0-6.0)	6.3 (3.2-9.4)	5.4 (3.7-7.1)
No. with infection/total No.	99 328/924 276	33 843/1 200 402	248 469/3 919 600	511 238/9 440 387
*P* value vs 2 doses^a^	.03			
*P* value vs 3 doses^a^	.26	.11		
*P* value vs unvaccinated^a^	.15	.01	.61	
**Other High-Risk HPV**^b^				
No. with infection	22	34	109	254
Weighted				
Prevalence (95% CI), %	22.7 (12.4-32.9)	28.1 (17.8-38.4)	27.2 (20.1-33.5)	25.2 (21.6-28.8)
No. with infection/total No.	209 527/924 276	337 309/1 200 402	1 064 296/3 919 600	2 378 757/9 440 387
*P* value vs 2 doses^a^	.39			
*P* value vs 3 doses^a^	.84	.89		
*P* value vs unvaccinated^a^	.61	.52	.61	

Abbreviation: HPV, human papillomavirus.

^a^ *P* values for survey weight adjusted Wald *F* test.

^b^ Other high-risk HPV includes types 35, 39, 51, 52, 56, 58, 59, and 68.

In adjusted analysis, the predicted probability of infection with HPV 6, 11, 16, or 18 was higher in unvaccinated women (7.4% [95% CI, 7.1%-7.7%]) compared with women who received 1 dose (2.3% [95% CI, 1.9%-2.8%), 2 doses (5.7 [95% CI, 5.1%-6.2%]), or 3 doses (3.1% [95% CI, 2.9%-3.4%]) (Table 2). Black women had a greater predicted probability (10.8%) of infection with HPV type 6, 11, 16, or 18 compared with white women (6.6%). The predicted probability was also higher for women with more than 5 lifetime male sexual partners (11.6%) than women with 0 to 5 lifetime male partners (3.3%).

**Table 2.** Difference in Predicted Probabilities of Infection With HPV Type 6, 11, 16, or 18 (4-Valent Vaccine–Type) by Risk Factor Among Women, National Health and Nutrition Examination Survey, 2009-2016

Risk Factor	Probability of Infection, % (95% CI)^a^	
	Predicted	Difference in Predicted Probability^b^
HPV vaccine dose(s)		
0 (Unvaccinated)	7.4 (7.1 to 7.7)	[Reference]
1	2.3 (1.9 to 2.8)	–5.0 (–5.6 to –4.5)
2	5.7 (5.1 to 6.2)	–1.7 (–2.4 to –0.1)
3	3.1 (2.9 to 3.4)	–4.3 (–4.6 to –4.0)
Race/ethnicity		
White	6.6 (6.3 to 6.8)	[Reference]
Black	10.8 (10.3 to 11.3)	4.2 (3.7 to 4.8)
Other^c^	5.9 (5.6 to 6.3)	–0.6 (–1.1 to –0.2)
Age at sexual debut, y		
<15	7.2 (6.9 to 7.5)	[Reference]
≥15	6.1 (5.8 to 6.4)	–1.1 (–1.5 to –0.2)
Lifetime No. of male sexual partners		
0-5	3.3 (3.1 to 3.4)	[Reference]
>5	11.6 (11.3 to 12.0)	8.3 (8.0 to 8.7)

Abbreviation: HPV, human papillomavirus.

^a^ Model was simultaneously adjusted for variables in the table and age as a linear term. The model included all 1315 women with nonmissing data on all listed variables.

^b^ Differences in predicted probability reflect the risk relative to the reference group adjusted for variables in the model and age as a linear term.

^c^ Mexican American, other Hispanic, or other races, including multiracial.

## Discussion

Our study suggests that US women who received 1 dose of the HPV vaccine may have gained similar protection against vaccine-type infections compared with those who received additional doses. These findings support previous observational studies and post hoc analyses of vaccine trials that demonstrated comparable effectiveness of 1 dose to 2 or 3 doses.^4^ The limitations of our study include a cross-sectional design that precluded us from evaluating the timing of HPV vaccination compared with potential exposure. Furthermore, any conclusion regarding efficacy by individual number of doses cannot be drawn given the self-reported immunization history that may be prone to recall bias.^6^ If ongoing trials confirm sufficient efficacy and sustained duration of protection from a single-dose regimen, vaccine initiation (as opposed to the series completion) will become a more achievable metric of population coverage.
